# Efficacy of mFOLFOX6 plus bevacizumab regimen in advanced colorectal cancer after deep hyperthermia: a single-center retrospective study

**DOI:** 10.3389/fonc.2023.1259713

**Published:** 2023-12-05

**Authors:** Guohu Han, Lihua Cui, Changchun Sun, Lijiang Yu, Shenzha Liu

**Affiliations:** Department of Oncology, Jingjiang People’s Hospital Affiliated with Yangzhou University, Jingjiang, China

**Keywords:** colorectal cancer, bevacizumab, deep hyperthermia, chemotherapy, treatment Karnofsky performance status

## Abstract

**Background:**

This study aimed to explore the clinical efficacy and safety of a modified FOLFOX6 (oxaliplatin + leucovorin + 5-fluorouracil) plus bevacizumab regimen after deep hyperthermia in advanced colorectal cancer.

**Methods:**

A total of 80 colorectal cancer patients treated at our hospital were selected as research subjects. According to the random number table method, patients were divided into a control group (mFOLFOX6 plus bevacizumab) and a combination group (mFOLFOX6 plus bevacizumab after deep hyperthermia treatment), with 40 patients in each group. After six cycles of treatment, the objective response rate (ORR), disease control rate (DCR), levels of serum tumor markers carcinoembryonic antigen (CEA), vascular epidermal growth factor (VEGF), Karnofsky performance status (KPS) scores, and the occurrence of adverse events were compared between the two groups.

**Results:**

After six cycles of treatment, the ORR in the combination group was higher than that in the control group, but the difference was not statistically significant (*P*>0.05). The DCR in the combination group was significantly higher than that in the control group (*P*<0.05). The serum CEA levels in the control and combination groups after treatment were significantly lower than those before treatment, and the serum CEA and VEGF levels in the combination group were significantly lower than those in the control group (all *P*<0.001). The KPS scores in both groups after treatment were higher than those before treatment, and the KPS scores in the combination group after treatment were significantly higher than those in the control group (all *P*<0.001). The incidence of fatigue and pain in the combination group was significantly lower than that in the control group (*P*<0.05).

**Conclusion:**

mFOLFOX6 plus bevacizumab after deep hyperthermia is effective in advanced colorectal cancer patients, which can effectively improve their quality of life, and the adverse events are controllable and tolerable. A randomized or prospective trial will be required to further prove these data and explore its potentiality, especially if compared to conventional treatment.

## Introduction

Colorectal cancer is the third most common cancer and the second most common cancer in terms of the number of people who develop cancer five years after diagnosis worldwide (1). The leading cause of death in colorectal cancer patients is distant metastases, with the most common sites of metastases being the liver and lung, followed by the lymph nodes, peritoneum, or brain, and 75% to 85% of patients cannot be treated surgically (2). The advancement of targeted therapeutic approaches, such as those focusing on inhibiting vascular endothelial growth factor (VEGF) signaling, BRAF and MEK pathways, as well as blocking epidermal growth factor receptor (EGFR) signaling, in addition to the integration of immunotherapy, in combination with conventional chemotherapy, has demonstrated enhanced efficacy in terms of both progression-free survival (PFS) and overall survival (OS) within certain specific subgroups of metastatic colorectal cancer patient populations (3). The utilization of the BRAF inhibitor encorafenib and the anti-EGFR agent cetuximab, either alone or in combination with the MEK inhibitor binimetinib, has exhibited remarkable enhancements in clinical efficacy while maintaining an acceptable level of toxicity, when compared to the use of standard chemotherapy regimens (4, 5). The overall enhanced responses of DNA mismatch repair-deficient (dMMR)/microsatellite instability-high (MSI-H) colorectal tumors to immunotherapy are primarily due to an increased immune cell infiltration compared to proficient/stable (MMRp/MSS) tumors (6). There is evidence that a dual immune checkpoint inhibitors (ICI) regimen has shown even greater benefits for dMMR/MSI-H metastatic colorectal cancer (7). Chemotherapy combined with targeted drugs is the first-line standard treatment for advanced colorectal cancer, but there is still a risk of progression after drug resistance, and adverse events in cancer patients are obvious (8, 9). Randomized controlled trials (RCT) have shown that patients receiving FOLFOX6 plus bevacizumab as first-line treatment for RAS mutant colorectal cancer had objective response rates of 54.5%, median progression-free survival 9.5 months, and median overall survival of 25.7 months; the incidences of proteinuria and hypertension were 9.9% and 8.3%, respectively (10). With the development of new antitumor technologies, patients are paying increasing attention to programs with good efficacy, low adverse events, and less trauma, such as hepatic artery embolization chemotherapy, microwave ablation, radiofrequency ablation, and deep hyperthermia. Because deep hyperthermia causes no trauma and does not increase the pain of patients, combined with conventional radiotherapy and chemotherapy, it can have a synergistic effect on a variety of anti-tumors (11–13). For more than four decades, the utilization of hyperthermia as a radiosensitizer or chemosensitizer has exhibited remarkable outcomes and currently demonstrates successful application when combined with radiotherapy or chemotherapy for the treatment of various tumor types. These include recurrent breast cancer, bladder cancer, cervical carcinoma, head and neck cancer, soft tissue sarcoma, and melanoma. Hyperthermia substantially enhances the effectiveness of both radiotherapy and chemotherapy, leading to significantly improved control of tumors and extended periods of disease-free survival (14). Simultaneously, individuals with locally advanced rectal cancer who experienced elevated cumulative temperatures related to hyperthermia exhibited a more robust rate of complete remission and tumor regression (15, 16). This study investigated the efficacy and adverse events of mFOLFOX6 combined with bevacizumab after deep hyperthermia in patients who could not be resected for advanced colorectal cancer, with the aim of providing new ideas for the treatment of such patients.

## Materials and methods

### Baseline data

**Figure 1:**
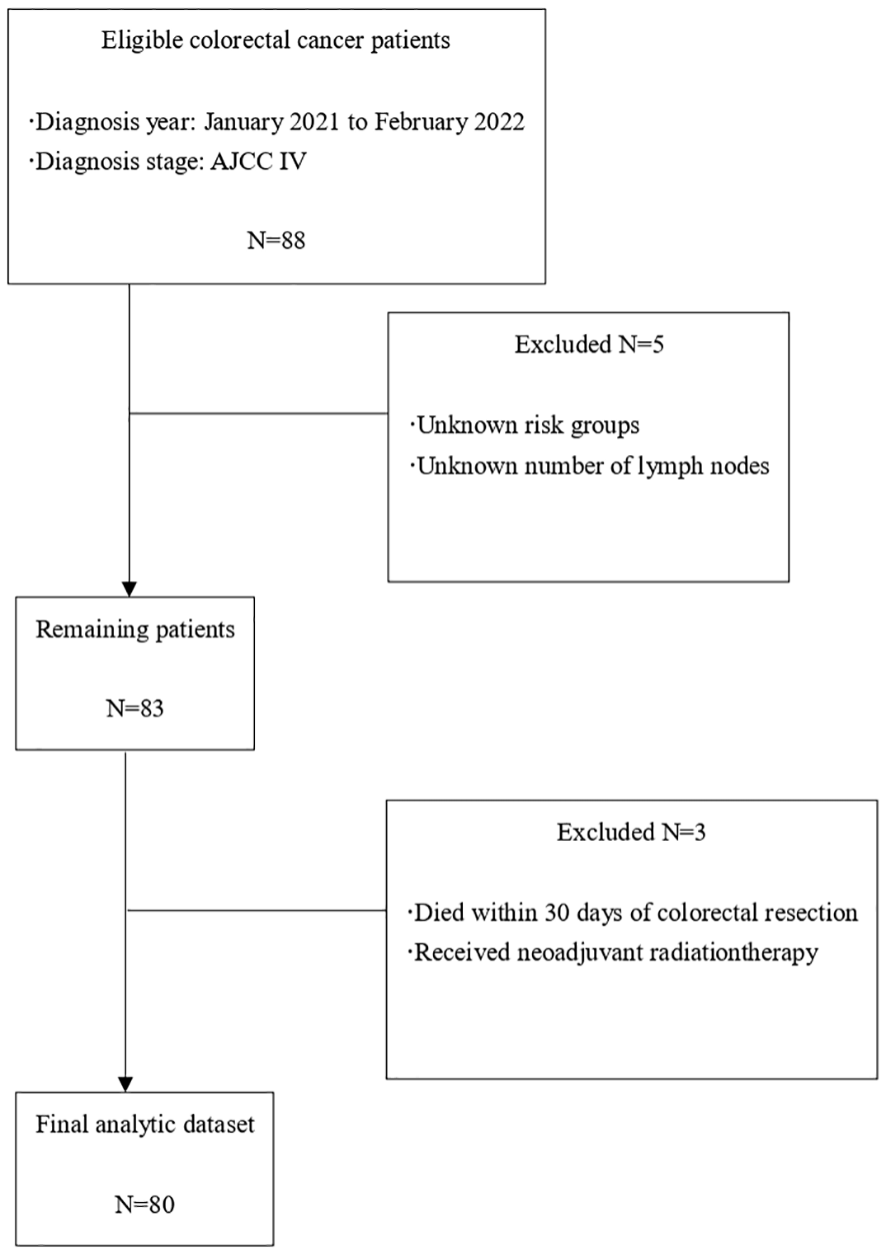
Flowchart of patient selection.

80 patients with colorectal cancer admitted to the seventh affiliated hospital of Yangzhou University from January 2021 to February 2022 were collected and divided into two groups according to the random number table method. Prior to enrollment, the two groups exhibited a relatively balanced distribution of clinical baseline information, including sex, age, site of primary tumor, differentiation degree, metastatic sites, and number of prior chemotherapy regimens. Detailed data can be found in **Figure 1** and **Table 1**, which illustrate the aforementioned characteristics. The Ethics Committee of Jingjiang People’s Hospital provided approval for this study (No. 2021-03-029). All subjects provided written informed consent, consistent with the Declaration of Helsinki.

**Table 1:** Baseline patient characteristics (*n* (%)).

Parameter (unit)	Control group (*n*=40)	Combination group (*n*=40)	*χ^2^*	*P*
Sex				
Male	24 (60.00)	18 (45.00)	1.805	0.179
Female	16 (40.00)	22 (55.00)		
Age (years)				
<65	25 (62.50)	28 (70.00)	0.503	0.478
≥65	15 (37.50)	12 (30.00)		
Site of primary tumor				
Rectum	13 (32.50)	12 (30.00)	0.521	0.771
Left hemicolon	18 (45.00)	21 (52.50)		
Right hemicolon	9 (22.50)	7 (17.50)		
Differentiation degree				
High differentiation	18 (45.00)	20 (50.00)	1.076	0.584
Moderate differentiation	19 (47.50)	15 (37.50)		
Low differentiation	3 (7.50)	5 (12.50)		
Liver metastasis				
Yes	11 (27.50)	9 (22.50)	0.267	0.606
No	29 (72.50)	31 (77.50)		
Brain metastasis				
Yes	0 (0.00)	1 (2.50)	1.013	0.314
No	40 (100.00)	39 (97.50)		
Number of prior regimens				
1	10 (25.00)	11 (27.50)	0.065	0.799
≥ 2	30 (75.00)	29 (72.50)		

### Inclusion and exclusion criteria

Inclusion criteria were as follows: ① all subjects must be diagnosed with advanced colorectal cancer by pathological and imaging examination, and asked for consultation with gastrointestinal surgery, hepatobiliary surgery, and neurosurgery, without surgical indications; ② KPS score ≥ 70; ③ estimated survival period ≥ 6 months; ④ at least one measurable solid lesion in the abdomen; ⑤ the results of liver and kidney function, blood regular test, and blood pressure were sound; ⑥ All patients and their relatives in this study signed the informed agreement and approved by the ethics committee of our hospital. The exclusion criteria were as follows:① patients with secondary primary tumor; ② patients with abnormal heart, liver, kidney and hematopoietic function and mental disorders; ③ advanced cachexia or severe basic medical diseases, abdominal skin collapse or infection; ④ anti-tumor history such as radiotherapy, chemotherapy, immunotherapy, and targeted therapy within 1 month; ⑤ patients with fever, ascites, thrombosis; ⑥ patients with gastrointestinal bleeding, perforation, or obstruction.

### Treatment methods

**Figure 2:**
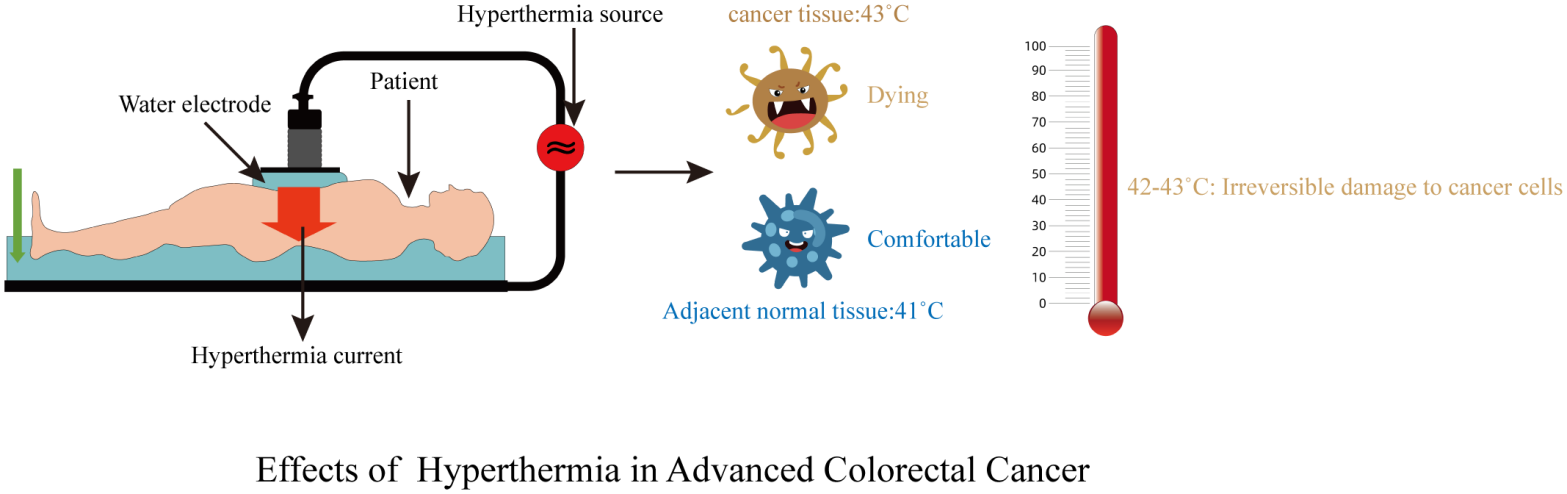
Treatment diagram.

**Figure 3:**
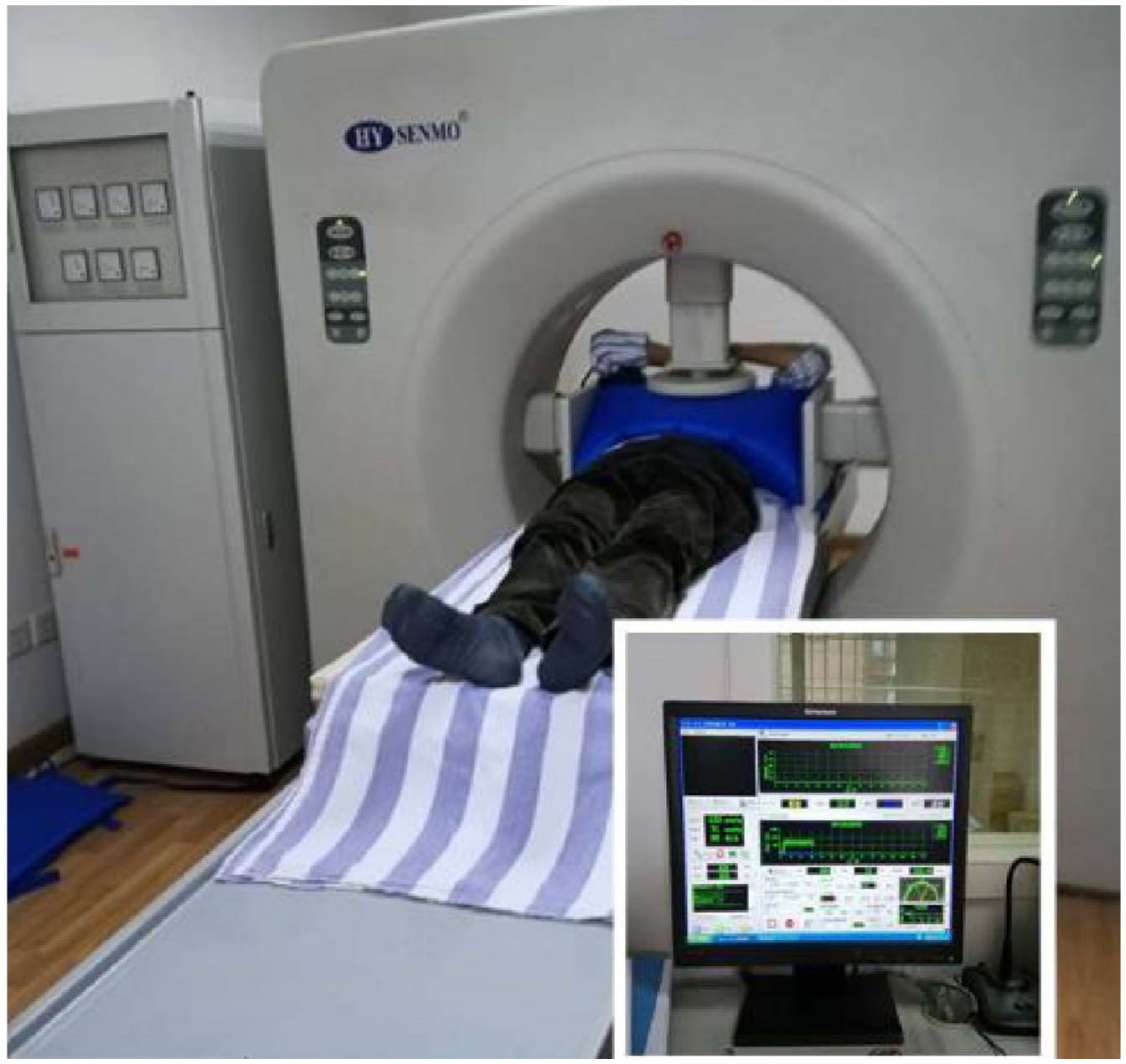
Treatment process.

For the control group, patients received bevacizumab once every 14 days (day 1:5 mg/kg) followed by mFOLFOX6 (day 1: oxaliplatin 85 mg/m^2^, leucovorin 400 mg/m^2^, and fluorouracil 400 mg/m^2^ intravenous bolus and then 2,400 mg/m^2^ more than 46 h continuous infusion) for 6 cycles of 14 days. In the combination group, all patients were administered a standardized treatment regimen consisting of mFOLFOX6 and bevacizumab following deep hyperthermia procedures. The tumor hyperthermia device utilized in this study was manufactured by Nanjing Hengpu Weiye Technology Co., LTD (Model NO. HY7000-1) and operated at a power range of 400-850 watts. The preset temperature was set at 42-43°C, and each treatment session had a duration of 40 minutes. The operation process of hyperthermia: Localization is performed according to CT, MR, and other imaging examinations, and the projection location of the tumor center is determined on the body surface. The temperature probe was fixed in the projection area on the body surface of the lesion center, and the temperature was monitored using a computer. The electrode plate was placed at the same level as the tumor center and was surrounded by sufficient water sacs to help dissipate heat. A computer control system was used to set the hyperthermia target area to completely cover the tumor. During treatment, the patients’ blood pressure, heart rate, and respiratory rate were closely monitored and sweating conditions were observed, as shown in **Figures 2**, **3**. The experimental protocol was approved by the ethics committee of Jingjiang People’s Hospital Affiliated with Yangzhou University.

### Outcome measures

Tumor markers included CEA and VEGF. CEA levels were measured using instrument-supported reagents on a Roche 2010 electrochemiluminescence spectrometer (Basel, Switzerland). Serum free VEGF was detected using an ELISA kit manufactured by Chemicon Company (USA), according to the manufacturer’s instructions. The normal reference values of serum CEA and VEGF were 0.0-3.5 ng/ml and 6.25-142.2 pg/ml, respectively.

Clinical efficacy was assessed utilizing PET/CT/MRI in accordance with the Response Evaluation Criteria in Solid Tumors (RECIST1.1) (17).

Complete remission (CR) was defined as the disappearance of all targeted lesions without the appearance of new lesions for a minimum of one month. Partial remission (PR) was characterized by a decrease in the total volume of the target lesions by at least 30% without the occurrence of new lesions for a minimum of one month. Progressive disease (PD) was identified by an increase in the total volume of the target lesions by at least 20% or the appearance of new lesions. Stable disease (SD) was defined as a lack of sufficient shrinkage to meet PR criteria or a lack of significant growth to meet PD criteria, with patients experiencing stable symptoms. Objective response rate (ORR) = (number of CR cases+ number of PR cases)/total number of cases × 100%. Disease control rate (DCR)= (number of CR cases+ number of PR cases+ number of SD cases)/total number of cases × 100.

The Karnofsky performance status (KPS) score was used to describe the patients’ quality of life between the two groups before and after treatment. The KPS scale is employed to assess a patient’s functional ability, with scores ranging from 0 to 100. A score of 100 indicates the patient’s full capability to carry out daily activities with no clinical evidence of disease, including signs or symptoms. Conversely, a score of 0 represents the patient’s decease. Higher scores indicate better quality of life (18). As shown in **Table 2**.

**Table 2:** Karnofsky performance status.

Conditions	Percentage	Comments
A: Able to perform normal daily activities and work. No special care is necessary.	100	Normal, without symptoms or signs of disease.
	90	Ability to perform normal daily activities, minor symptoms or signs of disease.
	80	perform normal daily activities with some effort, some symptoms or signs of disease.
B: Ability to live at home and take care of most personal needs. Assistance is needed to varying degrees.	70	Can take care of self, unable to do work or maintain normal daily activities. Such as cooking, playing football, and driving a car.
	60	Can take care of self the most of the time, but occasionally required considerable assistance.
	50	Required considerable assistance frequently.
C: Unable to take care of self. Hospital care is required. Disease can advance rapidly.	40	Disabled, requires medical care and assistance, in bed ≥50% of the time.
	30	Seriously disabled, unable to take care of themselves, almost always in bed.
	20	Seriously bedfast, hospitalization necessary, requiring active supportive treatment.
	10	Moribund, comatose or difficult to wake up.
	0	Dead.

Adverse events were assessed and graded based on the criteria outlined in the National Cancer Institute’s Common Terminology Criteria for Adverse Events version 4.0 (NCI-CTCAE v4.0).

### Statistical analysis

</u>Statistical analyses were conducted using SAS version 9.2 (SAS Institute, Inc., Cary, NC) and GraphPad Prism 8.02 (GraphPad Software, Inc.). The categorical data were expressed as (*n* (%)) and analyzed via the chi-square test and correction of the continuity chi-square test. The continuous data were reported as mean ± standard deviation (mean ± SD), and comparisons before and after treatment within the same group was assessed using paired samples *t* test. The comparison between mean ± SD of the two groups was performed using unpaired independent samples *t* test. A statistically significant difference was defined as *P* < 0.05 (2-sided).

## Results

### Comparative analysis of clinical efficacy before and after treatment between the two groups

After treatment, the control and combination groups showed 50.00% (20/40) and 70.00% (28/40), matter of ORR with no statistically significant difference (*P* > 0.05). The DCR in the combination group 90.00% (36/40) was significantly higher than that in the control group70.00% (28/40), with a statistically significant difference (*P*< 0.05). as shown in **Table 3**.

**Table 3:** Comparison of clinical efficacy of patients (*n* (%)).

Groups	*n*	CR	PR	SD	PD	ORR	DCR
Control group	40	3 (7.50)	17 (42.50)	8 (20.00)	12(30.00)	20 (50.00)	28 (70.00)
Combination group	40	2 (5.00)	26 (65.00)	8 (20.00)	4 (10.00)	28 (70.00)	36 (90.00)
*χ^2^*						3.333	5.000
*P*						0.068	0.025

CR, complete response; PD, progressive disease; PR, partial response; SD, stable disease; ORR, objective response rate; DCR, disease control rate.

### Comparative analysis of the levels of serum CEA, and VEGF before and after treatment between the two groups

The levels of serum CEA [(12.41 ± 1.29) μg/L *vs.* (21.67 ± 1.68) μg/L; (4.11 ± 1.36) μg/L *vs.* (22.02 ± 1.79) μg/L] and VEGF [(182.36 ± 13.42) pg/ml *vs.* (253.24 ± 11.06) ng/ml; (120.08 ± 14.19) ng/ml *vs.* (249.31 ± 12.14) ng/ml] in the control and combination groups after 6 cycles of treatment were significantly decreased compared with those before treatment, and the levels of serum CEA and VEGF in the combination group were significantly lower than those in the control group (all *P*< 0.001). as shown in **Tables 4**, **5**.

**Table 4:** Comparison of serum CEA expression level of patients (mean ± standard deviation).

Groups	*n*	Before treatment(μg/L)	After treatment(μg/L)	*t*	*P*
Control group	40	21.67 ± 1.68	12.41 ± 1.29	27.650	< 0.001
Combination group	40	22.02 ± 1.79	4.11 ± 1.36	50.387	< 0.001
*t*		0.902	28.004		
*P*		0.370	< 0.001		

CEA, carcinoembryonic antigen.

**Table 5:** Comparison of serum VEGF expression level of patients (mean ± standard deviation).

Groups	n	Before treatment(pg/ml)	After treatment(pg/ml)	*t*	*P*
Control group	40	253.24 ± 11.06	182.36 ± 13.42	25.778	< 0.001
Combination group	40	249.31 ± 12.14	120.08 ± 14.19	43.767	< 0.001
*t*		1.513	20.168		
*P*		0.134	< 0.001		

VEGF, Vascular endothelial growth factor.

### Comparative analysis of the changes of the KPS scores before and after treatment between the two groups

The KPS scores in the both groups after treatment were (82.19 ± 1.57) and (88.72 ± 1.62), respectively, which were higher than (75.81 ± 1.71) and (76.20 ± 1.80) before treatment, with statistically significant differences, and the KPS scores in the combination group after treatment were significantly higher than those in the control group (all *P*< 0.001). as shown in **Table 6**.

**Table 6:** Comparison of KPS scores of patients (mean ± standard deviation).

Groups	*n*	Before treatment	After treatment	*t*	*P*
Control group	40	75.81 ± 1.71	82.19 ± 1.57	17.382	< 0.001
Combination group	40	76.20 ± 1.80	88.72 ± 1.62	32.698	< 0.001
*t*		0.993	18.307		
*P*		0.324	< 0.001		

KPS, Karnofsky performance status.

### Comparative analysis of adverse events of patients in the two studied groups

The incidences of leucopenia, thrombocytopenia, anemia, nausea, vomiting, fever, burn, ascites, rash and gastrointestinal Hemorrhage in the two groups were no statistically significant differences (*P* > 0.05). The incidence of fatigue and pain in the combination group was significantly lower than that in the control group (*P*< 0.05). as shown in **Table 7**.

**Table 7:** Comparison of adverse events of patients (*n* (%)).

Adverse event	No. (%) of adverse events overall and by grade								
	Control group (*n*=40)				Combination group (*n*=40)				*P* value
	Grade				Grade				
	I	II	III	IV	I	II	III	IV	
Leucopenia	11 (27.5)	14 (35)	8 (20)	0 (0)	14 (35)	16 (40)	5 (12.5)	0 (0)	0.531
Thrombocytopenia	4 (10)	4 (10)	0 (0)	0 (0)	3 (7.5)	1 (2.5)	1 (2.5)	0 (0)	0.363
Anemia	8 (20)	6 (15)	1 (2.5)	2 (5)	4 (10)	9 (22.5)	2 (5)	0 (0)	0.648
Fatigue	20 (50)	7 (17.5)	0 (0)	0 (0)	11(27.5)	0 (0)	0 (0)	0 (0)	<0.001
Nausea	23 (57.5)	6 (15)	0 (0)	0 (0)	28 (70)	3 (7.5)	0 (0)	0 (0)	0.606
Vomiting	6 (15)	2 (5)	0 (0)	0 (0)	5 (12.5)	0 (0)	0 (0)	0 (0)	0.363
Fever	5 (12.5)	1 (2.5)	0 (0)	0 (0)	7 (17.5)	0 (0)	0 (0)	0 (0)	0.762
Pain	21 (52.5)	6 (15)	0 (0)	0 (0)	11 (27.5)	2 (5)	0 (0)	0 (0)	0.002
Burn	0 (0)	0 (0)	0 (0)	0 (0)	2 (5)	0 (0)	0 (0)	0 (0)	0.474
Ascites	7 (17.5)	1 (2.5)	1 (2.5)	0 (0)	6 (15)	4 (10)	0 (0)	0 (0)	0.793
Rash	3 (7.5)	0 (0)	0 (0)	0 (0)	2 (5)	2 (5)	0 (0)	0 (0)	1.000
Hemorrhage, GI	4 (10)	1 (2.5)	0 (0)	0 (0)	3 (7.5)	0 (0)	0 (0)	0 (0)	0.709

GI, gastrointestinal.

## Discussion

Currently, systemic chemotherapy and targeted therapy are the main treatments for inoperable advanced colorectal cancer. However, with the accumulation of drugs in the body, in addition to killing tumor cells, bone marrow hematopoietic cells, gastrointestinal mucosal cells, and other normal cells are also damaged, resulting in a series of adverse events such as anemia, agranulocytosis, nausea, vomiting, hair loss, and bleeding (19, 20). Several clinical studies on advanced colorectal cancer have shown that chemotherapy combined with bevacizumab can improve patient outcomes (21–23). A meta-analysis involving 700 subjects reported that 358 patients were treated with FOLFOX combined with bevacizumab, of which 53.35% achieved ORR and 82.96% achieved DCR, and 342 patients were treated with FOLFOX, 27.78% achieved ORR, and 63.74% achieved DCR. In terms of the ORR and DCR, the clinical efficacy of FOLFOX combined with bevacizumab was better than that of FOLFOX alone in the treatment of advanced colorectal cancer (24). Furthermore, a randomized controlled trial involving 3178 people was conducted to analyze adverse events, and it was found that gastrointestinal reactions were one of the most common adverse events, mainly manifested as nausea, vomiting, diarrhea, and anorexia. The incidence of gastrointestinal reactions was 24.33% in the FOLFOX combined with bevacizumab treatment group and 20.01% in the FOLFOX-only group (24). A preliminary investigation was conducted to assess the effectiveness of combining bevacizumab and FOLFOX-4 with deep electro-hyperthermia in previously untreated patients with metastatic colon cancer. The results of this combination therapy demonstrated significant disease control. Specifically, at timepoint-1 and timepoint-2, a DCR of 95% and 89.5% was observed, respectively (25). Another noteworthy finding of this study was the absence of significant toxicity associated with deep electro-hyperthermia treatment (25). It is suggested that hyperthermia combined with the FOLFOX6 regimen has a good effect in the treatment of advanced colorectal cancer, and adverse events can be tolerated.

In the present study, results showed that the ORR of the combined group was 70.00%, which was higher than that of the control group (50.00%) (*P* > 0.05); and The DCR of the combined group was 90.0%, which was higher than that of the control group (70.00%, *P*< 0.05). This indicates that the clinical efficacy of mFOLFOX6 combined with bevacizumab after deep hyperthermia was higher than that of mFOLFOX6 combined with bevacizumab alone. Its mechanism of action is that deep hyperthermia relies on the thermal sensitivity of tumor cells (26). On the one hand, high-temperature cytotoxicity acts on the tumor cell membrane and cytoskeleton, increasing the fluidity and permeability of the cell membrane, changing the tension of the cell membrane surface, breaking the internal and external balance of the cell, increasing the concentration of antitumor drugs in cells, accelerating cell apoptosis, and improving the ability to kill tumors. On the other hand, high temperatures induce protein denaturation and coagulative necrosis in localized tumor cells, resulting in detachment of tumor tissue, and consequential impairment of tumor cell synthesis and repair functions, and has negative thermal tolerance to deep hyperthermia, thus damaging cells and achieving tumor control (25, 26). With a decrease in tumor burden *in vivo*, the level of tumor markers in the peripheral blood will also decrease.

Serum tumor markers are associated with tumor diagnosis and disease progression. CEA and VEGF are the most common serum tumor markers of colorectal cancer (27). Elevated serum levels of CEA are commonly utilized for the diagnosis of colorectal cancer, as they are indicative of tumor invasion depth, lymph node metastasis, and tumor metastasis (28). In addition to CEA, a previous study showed that serum VEGF was highly positively correlated with lymph angiogenesis and tumor progression in patients with colorectal cancer (29). Bevacizumab exerts potent inhibition on the binding of VEGF to vascular endothelial growth factor receptor (VEGFR), effectively suppressing angiogenesis and impeding the proliferation and metastasis of tumor cells (30). One study showed that hyperthermia may have an anti-lymphangiogenic effect by inhibiting the expression of tumor VEGF, thereby inhibiting the lymphatic metastasis of cancer cells in tongue squamous cell carcinoma (31). Chemotherapy combined with hyperthermia was used in patients with lung cancer, and the serum CEA level in the combined treatment group was lower than that in the single-treatment group (32). This study showed that the levels of serum CEA and VEGF in the combined group were lower than those in the control group (all *P*< 0.05). Overall, hyperthermia may improve the prognosis of patients with advanced colorectal cancer.

This study suggests that there was no significant difference between the two groups in the incidence of hypertension, bleeding, bone marrow suppression, transaminase elevation, nausea, and vomiting (all *P* > 0.05), which was mainly grade I-III. Symptomatic treatment can be improved without affecting the next cycle of treatment. However, the incidence of fatigue and pain in the control group was significantly higher than that in the combined group (*P*< 0.05), which may be related to the fact that high temperatures help blood circulation and accelerate the discharge of metabolic waste, thus improving the quality of life and immune function of patients (33–35). Compared with the control treatment group, combination therapy was more effective and less toxic.

Future studies could compare this regimen with other emerging treatments for advanced colorectal cancer to establish relative efficacy and safety. Similar to the rationale behind using deep hyperthermia, CTLA-4 inhibitors might also contribute to reducing the side effects of chemotherapy by potentially requiring lower doses of chemotherapeutic agents due to the enhanced immune response (36, 37). Since hyperthermia can enhance the efficacy of chemotherapy and targeted therapies, there’s potential to explore how it interacts with CTLA-4 inhibitors (38, 39). Hyperthermia may alter the tumor microenvironment in a way that makes immunotherapy more effective (40). Indeed, CTLA-4, capecitabine, chemotherapy in colorectal cancer and immune checkpoint, immune checkpoint inhibitors and immunotherapy are crucial topics and should be given more attention in the future.

Although there is a lot of debate and controversy on the medical treatment of patients with advanced colorectal cancer, every new effective treatment that we explore, every new hypothesis that is put forward, whether it is accepted or rejected, gets us a bit closer to doing that we do. This study represents the pioneer utilization of a combination therapy approach involving chemotherapy, targeted therapy, and deep hyperthermia for cancer treatment. However, certain limitations are worth acknowledging. These include a small sample size, a relatively short treatment duration, and the absence of survival time observation during the initial design phase. As a result, further verification through larger-scale clinical trials and long-term efficacy assessments is necessary to substantiate these findings.

## Conclusion

In summary, mFOLFOX6 combined with bevacizumab after deep hyperthermia is effective in patients with advanced colorectal cancer, with good quality of life and controllable adverse events. Notably, the combination therapy leads to substantial reductions in tumor marker levels and significantly enhances the clinical response rate. With these favorable outcomes, its broader implementation holds promise and merits consideration. Consequently, additional randomized controlled trials and prospective studies are warranted to provide a more definitive assessment on this topic.

## Data availability statement

The original contributions presented in the study are included in the article/supplementary material. Further inquiries can be directed to the corresponding author.

## Ethics statement

The studies involving humans were approved by Ethics Committee of the Seventh Affiliated Hospital of Yangzhou University (No. 2021-03-029). The studies were conducted in accordance with the local legislation and institutional requirements. The participants provided their written informed consent to participate in this study. Written informed consent was obtained from the individual(s) for the publication of any potentially identifiable images or data included in this article.

## Author contributions

GH: Conceptualization, Formal Analysis, Investigation, Methodology, Project administration, Resources, Software, Supervision, Validation, Visualization, Writing – original draft, Writing – review & editing. LC: Conceptualization, Data curation, Investigation, Project administration, Software, Writing – original draft. CS: Data curation, Methodology, Software, Supervision, Validation, Visualization, Writing – review & editing. LY: Investigation, Methodology, Project administration, Resources, Supervision, Validation, Writing – review & editing. SL: Conceptualization, Data curation, Formal Analysis, Investigation, Methodology, Project administration, Resources, Validation, Visualization, Writing – review & editing.

## References

[1] Thanikachalam K Khan G. Colorectal cancer and nutrition. Nutrients (2019) 11 (1):164(1)–164(11). doi: 10.3390/nu11010164 30646512 PMC6357054

[2] Wang N Liu F Xi W Jiang J Xu Y Guan B. Development and validation of risk and prognostic nomograms for bone metastases in Chinese advanced colorectal cancer patients. Ann Transl Med (2021) 9(10):875. doi: 10.21037/atm-21-2550 34164509 PMC8184451

[3] Colle R Lonardi S Cachanado M Overman MJ Elez E Fakih M. BRAF V600E/RAS mutations and lynch syndrome in patients with MSI-H/dMMR metastatic colorectal cancer treated with immune checkpoint inhibitors. Oncologist. (2023) 28(9):771–9. doi: 10.1093/oncolo/oyad082 PMC10485382 37023721

[4] Ros J Baraibar I Sardo E Mulet N Salvà F Argilés G. BRAF, MEK and EGFR inhibition as treatment strategies in BRAF V600E metastatic colorectal cancer. Ther Adv Med Oncol (2021) 13:1758835921992974. doi: 10.1177/1758835921992974 33747149 PMC7903827

[5] Bond MJG Bolhuis K Loosveld OJL de Groot JWB Droogendijk H Helgason HH. First-line systemic treatment strategies in patients with initially unresectable colorectal cancer liver metastases (CAIRO5): an open-label, multicentre, randomised, controlled, phase 3 study from the Dutch Colorectal Cancer Group. Lancet Oncol (2023) 24(7):757–71. doi: 10.1016/S1470-2045(23)00219-X 37329889

[6] Llosa NJ Cruise M Tam A Wicks EC Hechenbleikner EM Taube JM. The vigorous immune microenvironment of microsatellite instable colon cancer is balanced by multiple counter-inhibitory checkpoints. Cancer Discovery (2015) 5(1):43–51. doi: 10.1158/2159-8290.CD-14-0863 25358689 PMC4293246

[7] Overman MJ Lonardi S Wong KYM Lenz HJ Gelsomino F Aglietta M. Durable clinical benefit with nivolumab plus ipilimumab in DNA mismatch repair-deficient/microsatellite instability-high metastatic colorectal cancer. J Clin Oncol (2018) 36(8):773–9. doi: 10.1200/JCO.2017.76.9901 29355075

[8] Mitbander UB Geer MJ Taxbro K Horowitz JK Zhang Q O'Malley ME. Patterns of use and outcomes of peripherally inserted central catheters in hospitalized patients with solid tumors: A multicenter study. Cancer. (2022) 128(20):3681–90. doi: 10.1002/cncr.34410 35943390

[9] Zhang JG Wang H Gu XF Wang XY Wang WJ Du LB. Status and associated factors of cross-regional healthcare-seeking among patients with advanced colorectal cancer in China: a multicenter cross-sectional study. Ann Transl Med (2022) 10(6):342. doi: 10.21037/atm-22-1003 35433943 PMC9011287

[10] Tang W Ren L Liu T Ye Q Wei Y He G. Bevacizumab Plus mFOLFOX6 Versus mFOLFOX6 Alone as First-Line Treatment for RAS Mutant Unresectable Colorectal Liver-Limited Metastases: The BECOME Randomized Controlled Trial. J Clin Oncol (2020) 38(27):3175–84. doi: 10.1200/JCO.20.00174 32749938

[11] Kroesen M van Holthe N Sumser K Chitu D Vernhout R Verduijn G. Feasibility, SAR distribution, and clinical outcome upon reirradiation and deep hyperthermia using the hypercollar3D in head and neck cancer patients. Cancers (Basel). (2021) 13(23):6149. doi: 10.3390/cancers13236149 34885258 PMC8656471

[12] Wen YC Lee LM Lin YW Syu SH Lin KH Fan YC. Loco-regional deep hyperthermia combined with intravesical Mitomycin instillation reduces the recurrence of non-muscle invasive papillary bladder cancer. Int J Hyperthermia. (2021) 38(1):1627–32. doi: 10.1080/02656736 34775895

[13] Franckena M Stalpers LJ Koper PC Wiggenraad RG Hoogenraad WJ van Dijk JD. Long-term improvement in treatment outcome after radiotherapy and hyperthermia in locoregionally advanced cervix cancer: an update of the Dutch Deep Hyperthermia Trial. Int J Radiat Oncol Biol Phys (2008) 70(4):1176–82. doi: 10.1016/j.ijrobp.2007.07.2348 17881144

[14] Datta NR Ordóñez SG Gaipl US Paulides MM Crezee H Gellermann J. Local hyperthermia combined with radiotherapy and-/or chemotherapy: recent advances and promises for the future. Cancer Treat Rev (2015) 41(9):742–53. doi: 10.1016/j.ctrv.2015.05.009 26051911

[15] Gani C Lamprecht U Ziegler A Moll M Gellermann J Heinrich V. Deep regional hyperthermia with preoperative radiochemotherapy in locally advanced rectal cancer, a prospective phase II trial. Radiother Oncol (2021) 159:155–60. doi: 10.1016/j.radonc.2021.03.011 33741467

[16] Ott OJ Gani C Lindner LH Schmidt M Lamprecht U Abdel-Rahman S. Neoadjuvant chemoradiation combined with regional hyperthermia in locally advanced or recurrent rectal cancer. Cancers (Basel). (2021) 13(6):1279. doi: 10.3390/cancers13061279 33805731 PMC8001688

[17] Zhou M Zhang C Nie J Sun Y Xu Y Wu F. Response Evaluation and Survival Prediction Following PD-1 Inhibitor in Patients With Advanced Hepatocellular Carcinoma: Comparison of the RECIST 1.1, iRECIST, and mRECIST Criteria. Front Oncol (2021) 11:764189. doi: 10.3389/fonc.2021.764189 34956885 PMC8697350

[18] Péus D Newcomb N Hofer S. Appraisal of the Karnofsky Performance Status and proposal of a simple algorithmic system for its evaluation. BMC Med Inform Decis Mak. (2013) 13:72. doi: 10.1186/1472-6947-13-72 23870327 PMC3722041

[19] Holmøy T Fevang B Olsen DB Spigset O Bø L. Adverse events with fatal outcome associated with alemtuzumab treatment in multiple sclerosis. BMC Res Notes. (2019) 12(1):497. doi: 10.1186/s13104-019-4507-6 31405369 PMC6689881

[20] Perger L Bürgi U Fattinger K. Pharmacotherapy of hyperthyreosis–adverse drug reactions. Ther Umsch. (2011) 68(6):303–8. doi: 10.1024/0040-5930/a000169 21656488

[21] Kashiwa M Matsushita R. Comparative cost-effectiveness of aflibercept and ramucirumab in combination with irinotecan and fluorouracil-based therapy for the second-line treatment of metastatic colorectal cancer in Japan. Clin Ther (2020) 42(7):1361–75. doi: 10.1016/j.clinthera.2020.05.013 32616433

[22] Loupakis F Cremolini C Masi G Lonardi S Zagonel V Salvatore L. Initial therapy with FOLFOXIRI and bevacizumab for metastatic colorectal cancer. N Engl J Med (2014) 371(17):1609–18. doi: 10.1056/NEJMoa1403108 25337750

[23] Tebbutt NC Wilson K Gebski VJ Cummins MM Zannino D van Hazel GA. Capecitabine, bevacizumab, and mitomycin in first-line treatment of metastatic colorectal cancer: results of the Australasian Gastrointestinal Trials Group Randomized Phase III MAX Study. J Clin Oncol (2010) 28(19):3191–8. doi: 10.1200/JCO.2009.27.7723 20516443

[24] Zhang H You J Liu W Chen D Zhang S Wang X. The efficacy and safety of bevacizumab combined with FOLFOX regimen in the treatment of advanced colorectal cancer: a systematic review and meta-analysis. Med (Baltimore). (2021) 100(30):e26714. doi: 10.1097/MD.0000000000026714 PMC8322501 34397704

[25] Ranieri G Laface C Laforgia M De Summa S Porcelli M Macina F. Bevacizumab plus FOLFOX-4 combined with deep electro-hyperthermia as first-line therapy in metastatic colon cancer: a pilot study. Front Oncol (2020) 10:590707. doi: 10.3389/fonc.2020.590707 33224885 PMC7670056

[26] Wust P Hildebrandt B Sreenivasa G Rau B Gellermann J Riess H. Hyperthermia in combined treatment of cancer. Lancet Oncol (2002) 3(8):487–97. doi: 10.1016/s1470-2045(02)00818-5 12147435

[27] Wang G Wang Y Yang X Zhang Y Lu Y Li Y. The expression and diagnostic value of serum levels of EphA2 and VEGF-A in patients with colorectal cancer. Cancer biomark (2021) 31(4):399–408. doi: 10.3233/CBM-201745 34092605 PMC12500029

[28] You W Yan L Cai Z Xie L Sheng N Wang G. Clinical significances of positive postoperative serum CEA and post-preoperative CEA increment in stage II and III colorectal cancer: a multicenter retrospective study. Front Oncol (2020) 10:671. doi: 10.3389/fonc.2020.00671 32509572 PMC7251078

[29] Dan LA Werdyani S Xu J Shestopaloff K Hyde A Dicks E. No associations of a set of SNPs in the Vascular Endothelial Growth Factor (VEGF) and Matrix Metalloproteinase (MMP) genes with survival of colorectal cancer patients. Cancer Med (2016) 5(9):2221–31. doi: 10.1002/cam4.796 PMC5055182 27334288

[30] Lee MS Ryoo BY Hsu CH Numata K Stein S Verret W. Atezolizumab with or without bevacizumab in unresectable hepatocellular carcinoma (GO30140): an open-label, multicentre, phase 1b study. Lancet Oncol (2020) 21(6):808–20. doi: 10.1016/S1470-2045(20)30156-X 32502443

[31] Liang X Zhou H Liu X He Y Tang Y Zhu G. Effect of local hyperthermia on lymphangiogenic factors VEGF-C and -D in a nude mouse xenograft model of tongue squamous cell carcinoma. Oral Oncol (2010) 46(2):111–5. doi: 10.1016/j.oraloncology.2009.12.001 20036606

[32] Zhou L Zhang T Sun Y Fan R Xu L Yue S. Effect of preoperative infusion chemotherapy combined with hyperthermia on sPD-L1 and CEA levels and overall survival of elderly patients undergoing radical resection of lung cancer. J BUON. (2019) 24(2):572–7. 31128008

[33] Zheng X Xu W Feng H Cao K. High and low temperature performance and fatigue properties of silica fume/SBS compound modified asphalt. Materials (Basel). (2020) 13(19):4446. doi: 10.3390/ma13194446 33036436 PMC7579355

[34] Luo L Huang JH Liu DL Jiang SG Zhou FL Jiang S. Transcriptome reveals the important role of metabolic imbalances, immune disorders and apoptosis in the treatment of Procambarus clarkii at super high temperature. Comp Biochem Physiol Part D Genomics Proteomics. (2021) 37:100781. doi: 10.1016/j.cbd.2020.100781 33316578

[35] Chang M Hou Z Wang M Li C Lin J. Recent advances in hyperthermia therapy-based synergistic immunotherapy. Adv Mater (2021) 33(4):e2004788. doi: 10.1002/adma.202004788 33289219

[36] Ludford K Ho WJ Thomas JV Raghav KPS Murphy MB Fleming ND. Neoadjuvant pembrolizumab in localized microsatellite instability high/deficient mismatch repair solid tumors. J Clin Oncol (2023) 41(12):2181–90. doi: 10.1200/JCO.22.01351 PMC10489404 36623241

[37] Sun C Yin M Cheng Y Kuang Z Liu X Wang G. Novel small-molecule PD-L1 inhibitor induces PD-L1 internalization and optimizes the immune microenvironment. J Med Chem (2023) 66(3):2064–83. doi: 10.1021/acs.jmedchem.2c01801 36579489

[38] Li Y Ju M Miao Y Zhao L Xing L Wei M. Advancement of anti-LAG-3 in cancer therapy. FASEB J (2023) 37(11):e23236. doi: 10.1096/fj.202301018R 37846808

[39] Guo S Feng J Li Z Yang S Qiu X Xu Y. Improved cancer immunotherapy strategies by nanomedicine. Wiley Interdiscip Rev Nanomed Nanobiotechnol. (2023) 15(3):e1873. doi: 10.1002/wnan.1873 36576112

[40] Wahab R Hasan MM Azam Z Grippo PJ Al-Hilal TA. The role of coagulome in the tumor immune microenvironment. Adv Drug Delivery Rev (2023) 200:115027. doi: 10.1016/j.addr.2023.115027 PMC11099942 37517779

